# Which egg features predict egg rejection responses in American robins? Replicating Rothstein's (1982) study

**DOI:** 10.1002/ece3.3759

**Published:** 2018-01-05

**Authors:** Alec B. Luro, Branislav Igic, Rebecca Croston, Analía V. López, Matthew D. Shawkey, Mark E. Hauber

**Affiliations:** ^1^ Department of Animal Biology School of Integrative Biology University of Illinois Urbana‐Champaign IL USA; ^2^ Division of Ecology & Evolution Research School of Biology Australian National University Canberra ACT Australia; ^3^ U.S. Geological Survey Western Ecological Research Center Dixon CA USA; ^4^ Departamento de Ecología, Genética y Evolución Facultad de Ciencias Exactas y Naturales Universidad de Buenos Aires Buenos Aires Argentina; ^5^ Department of Biology, Evolution and Optics of Nanostructures Group University of Ghent Ghent Belgium

**Keywords:** avian brood parasitism, egg recognition, *Molothrus ater*, *Turdus migratorius*

## Abstract

Rothstein (Behavioral Ecology and Sociobiology, 11, 1982, 229) was one of the first comprehensive studies to examine how different egg features influence egg rejection behaviors of avian brood parasite–hosts. The methods and conclusions of Rothstein (1982) laid the foundation for subsequent experimental brood parasitism studies over the past thirty years, but its results have never been evaluated with replication. Here, we partially replicated Rothstein's (1982) experiments using parallel artificial model egg treatments to simulate cowbird (*Molothrus ater*) parasitism in American robin (*Turdus migratorius*) nests. We compared our data with those of Rothstein (1982) and confirmed most of its original findings: (1) robins reject model eggs that differ from the appearance of a natural robin egg toward that of a natural cowbird egg in background color, size, and maculation; (2) rejection responses were best predicted by model egg background color; and (3) model eggs differing by two or more features from natural robin eggs were more likely to be rejected than model eggs differing by one feature alone. In contrast with Rothstein's (1982) conclusion that American robin egg recognition is not specifically tuned toward rejection of brown‐headed cowbird eggs, we argue that our results and those of other recent studies of robin egg rejection suggest a discrimination bias toward rejection of cowbird eggs. Future work on egg recognition will benefit from utilizing a range of model eggs varying continuously in background color, maculation patterning, and size in combination with avian visual modeling, rather than using model eggs which vary only discretely.

## 1 INTRODUCTION

Reproducibility is a central concern of the modern scientific approach and paramount to research progress (Baker, 2016; Kelly, 2006; Nakagawa & Parker, 2015). Confidence in empirical conclusions depends on successful replication of experimental data and results; that is, if a discoverable general pattern exists in nature, then it should be measurable, consistent, and reproducible by multiple independent laboratories (Simons, 2014). Here, we reevaluate critical and impactful findings of one of the first comprehensive studies on brood parasite egg rejection by avian hosts that examined how different features of foreign eggs influence hosts’ rejection decisions.

Interspecific avian brood parasites do not build their own nests or raise their own offspring; instead, they lay their eggs in the nests of host species (Davies, 2000). Hosts may accept the brood parasite's egg and raise unrelated offspring at a cost to their own fitness (Hauber, 2003), or recognize the foreign egg and remove it from the nest (Payne, 1977). Stephen Rothstein's dissertation at Yale University, the many resulting papers, and especially his landmark *Behavioral Ecology and Sociobiology* 1982 study of egg rejection by American robins (*Turdus migratorius*; hereafter: robin) and gray catbirds (*Dumetella carolinensis*) (Rothstein, 1982), paved the way for subsequent studies focusing on characterizing robins’ and other host species’ abilities to discriminate their own eggs from those of their respective brood parasites (or from foreign eggs in general; Hauber et al., 2015). Through careful design of artificial model eggs constructed from plaster of Paris and painted with acrylic and latex paints, Rothstein (1982) separated the relative influences of egg size, background color, and spotting (or “maculation”) on robin and gray catbird egg rejection responses. Egg rejection behavior in robins likely evolved as a defense against brood parasitism by the mostly sympatric brown‐headed cowbird (*Molothrus ater*; hereafter: cowbird), an obligate interspecific brood parasite (Abernathy & Peer, 2015; Briskie, Sealy, & Hobson, 1992; Croston & Hauber, 2014, 2015a; Friedmann, 1929; Kuehn, Peer, & Rothstein, 2014; Lang, Bollinger, & Peer, 2014; Rothstein, 1975a). Notably, Rothstein (1982) found that robins respond most strongly to experimental model eggs that deviate from their own eggs’ appearance and toward that of a cowbird egg in at least two of the three features tested (i.e., background color, maculation, and size) and respond only weakly to experimental eggs that differ from their own eggs by one feature alone.

Here, we set out to replicate Rothstein's (1982) experiments using a parallel set of artificial model eggs to reexamine the relative influence of discrete differences in model egg background color, maculation, and size on robin egg rejection decisions. We conducted a partial replication (for replication type definitions, see Kelly, 2006; Nakagawa & Parker, 2015) of Rothstein's (1982) experimental methods, combined data from our experiments with those of Rothstein (1982), and analyzed which egg features predict robin egg rejection responses with an information‐theoretic statistical approach using generalized linear mixed models (Bolker et al., 2009; Burnham & Anderson, 2002; Symonds & Moussalli, 2011).

## 2 METHODS

### 2.1 Data and model eggs

**Figure 1:**
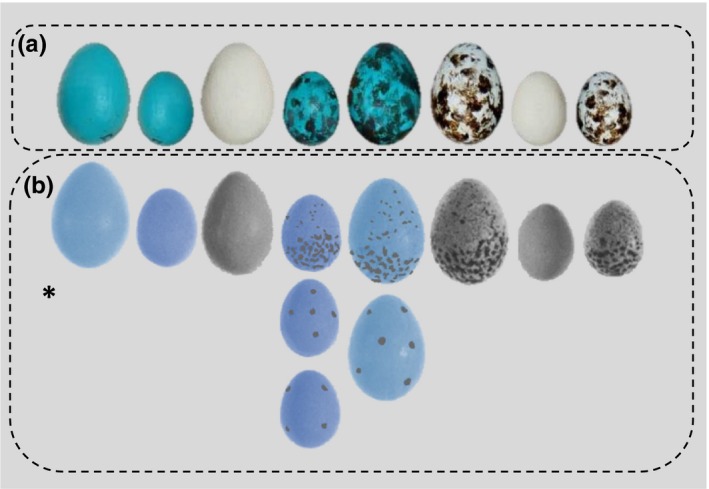
Artificial eggs used in parasitism experiments of (a) current study and (b) Rothstein (1982). Artificial eggs were regarded as the following treatments in analyses, from left to right: large mimetic (*LM*), small mimetic (*SM*), large beige (*LB*), small mimetic spotted (*SMS*), large mimetic spotted (*LMS*), large beige spotted (*LBS*), small beige (*SB*), and small beige spotted (*SBS*). Data for Rothstein's (1982) *SMS* and *LMS* eggs were aggregated for the respective three and two egg treatments shown in (b). Sizes of eggs used in both studies (a,b) are accurate relative to one another, within each respective study. (a,b) *Images of Rothstein's (1982) artificial eggs were adapted from Rothstein's (1982) Figure 1 depiction of model eggs. Images were recolored for illustrative purposes and do not represent actual color of eggs used in Rothstein (1982)

We extracted the published model‐type level egg rejection data from Rothstein (1982) of robins’ responses to various experimental eggs placed into their nests (for data source: see Rothstein, 1982; fig. 3) to combine with our own data (for data, see Table S1). Artificial model eggs of both studies were specifically designed to represent a discrete spectrum of egg sizes, background colors, and maculation pattern combinations ranging in appearance from a robin egg to a cowbird egg (Figure 1). We recoded and binned model egg data from Rothstein (1982) as follows (original coding indicated by *R′82* subscript): *W*_(*R′82*)_ = white/beige, cowbird‐mimetic background color; *S*_(*R′82*)_ = small, cowbird‐sized; *M*_(*R′82*)_ and *P*_(*R′82*)_ = maculated, mimetic cowbird‐colored spotting. Experimental model eggs’ features of both Rothstein (1982) and our study are as follows: *size* = robin‐sized (*L*) or cowbird‐sized (*S*); *background color* = robin blue‐green colored (*M*) or cowbird white/beige colored (*B*); *maculation* = spotted (*S*) or immaculate (absence of *S* code). Model eggs of both Rothstein (1982) and this study were constructed of plaster of Paris and colored using acrylic or latex paints; for painting, size, and manufacture details of our model eggs, see Croston and Hauber (2014), “BHCO ground” and “AMRO (mimetic) ground” colors.

Small cowbird‐sized and large robin‐sized model eggs used by this study resembled the dimensions and masses of natural cowbird (2.6–3.4 g, 21 × 16 mm) and robin eggs (4.2–8.4 g, 31 × 21 mm). Model eggs of Rothstein (1982) also resembled the dimensions of natural cowbird and robin eggs, but were estimated to be 10%–17% heavier than natural eggs (see Rothstein, 1975b). Model egg mass can significantly affect host egg rejection responses, because heavier eggs are less likely to be successfully rejected (Ruiz‐Raya, Soler, Sánchez‐Pérez, & Ibáñez‐Álamo, 2015). However, robins are adept grasp ejectors (Rasmussen, Sealy, & Underwood, 2009; Rasmussen, Underwood, & Sealy, 2010), capable of removing model eggs within the range of natural robin egg size and mass (Underwood & Sealy, 2006a; Rasmussen et al., 2009; personal observation 2012). The European blackbird (*Turdus merula*), a congener of American robins with similar body size and bill morphology, can remove model eggs weighing at least 10 g (Ruiz‐Raya et al., 2015). Therefore, it was unlikely that a potential disparity between masses of model eggs used in our experiments versus Rothstein's (1982) would significantly affect the results.

### 2.2 Subjects and study areas

Robins are a rejecter host species of cowbirds (Rothstein, 1975a); robin populations sympatric with cowbirds reject about 100% of natural cowbird eggs that are deposited into their nests (Briskie et al., 1992). Rothstein (1982) tested nesting robins in Connecticut and Michigan, USA, between the years of 1966 and 70 (*N* = 93 total parasitism trials). We conducted a total of *N* = 125 experimental parasitism trials in Ithaca, NY, from 2010 to 2014 (*N* = 109) and Urbana, IL, in 2015 (*N* = 16). Although there may be some variation in egg rejection behavior between American robin populations that are allopatric versus sympatric with cowbirds (Briskie et al., 1992), experimental brood parasitism data from all populations of robins studied by Rothstein (1982) and this study are comparable because cowbirds have been sympatric with robins across all locations where artificial parasitism experiments took place since the U.S. Geological Survey began its North American Breeding Bird Survey in 1966 (Sauer et al., 2014).

### 2.3 Experimental parasitism

For Rothstein's (1982) data, we combined data of robins’ responses to model eggs in both the nesting (i.e., laying) and incubation stages (i.e., stages 1 and 2, respectively) because our data also came from both nesting periods unseparated. The nest stage at either the start or end of an experimental parasitism trial does not significantly influence robins’ responses to the model eggs placed in their nests at the Ithaca and Urbana study sites where our own experiments took place (Croston & Hauber, 2014; Luro & Hauber, 2017). Likewise, Rothstein (1982) acknowledged results would have only been slightly different if data from both nesting stages were combined.

For our own experiments, we followed the experimental brood parasitism methods of Igic et al. (2015). In brief, a model egg was placed into an active robin nest (i.e., nest containing one or more eggs) found in either the laying or incubating stage. Unlike Rothstein (1982), we did not remove a single robin egg from the nest and replace it with an experimental model egg, because the removal of natural robin eggs from the nest does not affect robins’ responses to model eggs placed in the nest (Briskie et al., 1992). Furthermore, cowbirds may not always remove a host egg before or after parasitizing a nest (Scott, 1977; Sealy, 1992). Therefore, the insertion of a foreign egg into a robin's nest alone, without removing a robin's own egg, is sufficient to simulate natural cowbird parasitism for this host species.

For both our and Rothstein's (1982) data, responses to model eggs were recorded as rejections if the model egg disappeared from the nest within 5 days from the day it was inserted into the nest. If the model egg remained in the nest after 5 days, the robin's response was recorded as an acceptance. If the nest was deemed abandoned (or deserted), eggs in the nest hatched, or the nest was depredated during the experimental period, the experimental trial was ended and excluded from the analyses. For a more detailed explanation of our experimental parasitism procedures on robins using plaster of Paris model eggs, see Croston and Hauber (2014) and Aidala, Croston, Schwartz, Tong, and Hauber (2015). Critically, these studies found no effect of repeated parasitism and nesting stage (laying vs. incubation) on egg rejection rates by American robins. Data from our own artificial parasitism experiments could not be collected blindly because our study involved observation of wild robin nests in the field.

### 2.4 Statistical analyses

To assess the relative influences of experimental egg size, background color, and maculation on robins’ egg rejection responses, we used generalized linear mixed models (GLMMs), constructed with the glmer function and fitted with Laplace approximations and binomial logit distributions using the lme4 package (Bates, Maechler, Bolker, & Walker, 2015) in combination with model averaging. All analyses were conducted in R v.3.2.4 (R Core Team 2016).

Initially, to assess whether combining of our own data with Rothstein's (1982) would be appropriate, we first analyzed a set of GLMMs using the MuMIn package with study ID (i.e., Rothstein, 1982 or this study's experimental data), along with model egg features and of all their interactions with study as fixed effects. Then, we selected the best models (see below) and calculated model‐averaged effect estimates and their 95% confidence intervals for all predictors included in the top models (the procedure for best model selection and model average effect estimates are explained below).

After finding no significant differences between Rothstein's (1982) and our own data (see Results), we then analyzed a new set of GLMMs which accounted for variation in robins’ rejection behaviors across the two studies by setting study ID as a random effect. For our global GLMM, the binary response variable was the rejection/acceptance of the artificial model egg, random effects included nest ID and study ID, and fixed effects included the model egg color (background blue‐green robin or white–beige cowbird), maculation (spotted or immaculate), size (robin‐sized or cowbird‐sized), and the interactions among all three model egg features. We ran all possible combinations of predictors included in the global GLMM as model iterations using the dredge function from the MuMIn package (Bartoń, 2016) and selected the best models using Akaike's information criteria corrected for sample size (AICc), setting a cutoff to include the fewest number of models whose sum of Akaike weights (*w*_i_) is greater than *w*_i_ = 0.95 (Burnham & Anderson, 2002; Wagenmakers & Farrell, 2004). Finally, we calculated model‐averaged effect estimates and 95% confidence intervals for all predictors included in the three final best models (Table 1).

**Table 1:** Model selection table for the three top models from all candidate models (*N* = 16) testing the effects of foreign egg color, maculation, and size on egg rejection decisions by American robins

Model	Model parameters							*df*	logLik	AICc	∆*i*	*w*_i_
	Color	Maculation	Size	Color × maculation	Color × size	Maculation × size	Color × maculation × size					
1	+	+	+		+			7	−83.51	181.55	0	0.48
2	+	+	+		+	+		8	−82.79	182.27	0.72	0.33
3	+	+	+	+	+			8	−83.36	183.40	1.85	0.19

For all models, Nest ID and Study ID (i.e., this study or Rothstein, 1982) were included as random effects. Top models were selected as the models whose cumulative sum of AICc weights (*w*_i_) > 0.95. ∆*i* = AICc(*i*) − AICc(min).

## 3 RESULTS

### 3.1 Comparison between studies

We did not find a significant difference in robins’ rejection responses between Rothstein (1982) and our own experiments (Rothstein, 1982 vs. current study log‐odds: −0.65, 95% CI = [−1.44, 0.15]). We also did not find any significant effects of interactions between study ID and the size, background color, or maculation pattern of the artificial model eggs (all study ID interaction variables’ model‐averaged effect estimate 95% CIs overlapped 0). Therefore, we accounted for variance in robins’ responses attributable to study ID by including study ID as a random effect in our final set of GLMMs.

### 3.2 Rejection responses toward specific model egg treatments

**Figure 2:**
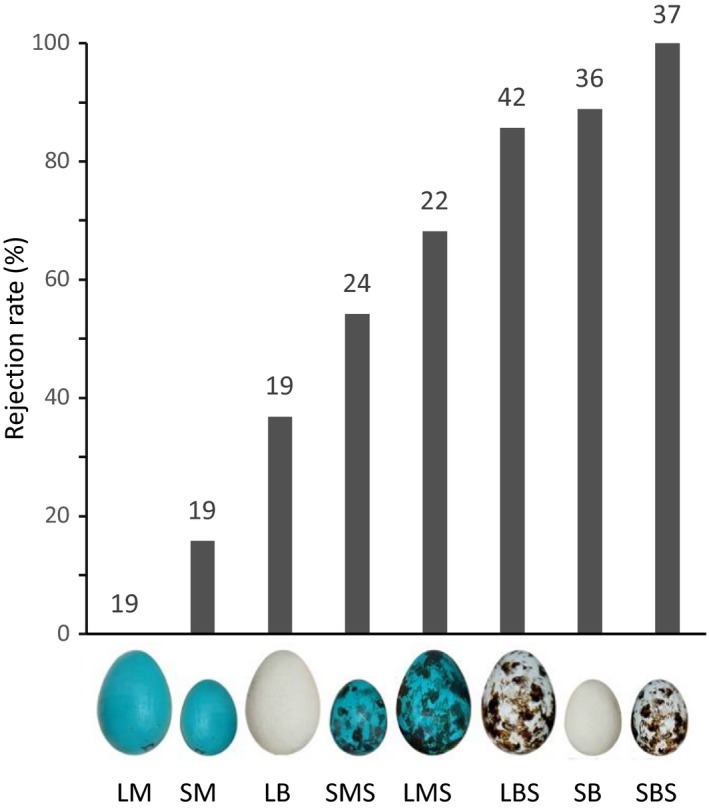
Rejection rates of experimental eggs placed in American robin nests. Data used are combined from this study and Rothstein (1982). Sample sizes of the number of trials for each treatment are listed above bars. Experimental egg treatment codes are in order of size (*L* = large robin‐sized, *S* = small cowbird‐sized), color (*M* = mimetic robin, *B* = beige cowbird), and maculation (*S* = spotted)

Model eggs with features resembling those of natural brown‐headed cowbird eggs (beige background color, small size, and maculation) were rejected at higher rates than model eggs with features resembling those of natural robin eggs (blue‐green background color, large size, and immaculate) (Figure 2). Robin egg rejection responses were best predicted by model egg background color (beige vs. blue‐green log‐odds: −3.80, 95% CI = [−5.62, −1.98]) and maculation (immaculate vs. spotted log‐odds: 3.4, 95% CI = [1.66, 5.15]), but also by model egg size (large vs. small log‐odds: 2.23, 95% CI = [0.70, 3.77]) (Tables 1 and 2). The effect of robin (large) versus cowbird‐sized (small) eggs was different for beige versus blue eggs (interaction between egg size and background color, log‐odds: −3.33, 95% CI = [−5.89, −0.77]; Figure 2). The effect of model egg maculation on rejection probability did not vary between the two egg sizes (interaction between egg size and maculation, log‐odds: −0.45, 95% CI = [−3.60, 0.91]) nor between egg background colors (interaction between egg background color and maculation, log‐odds: 0.12, 95% CI = [−1.7, 2.95]) (Tables 1 and 2).

**Table 2:** Model‐averaged mean effect estimates (log‐odds) and 95% confidence intervals for the influence of model egg parameters on egg rejection outcomes in American robins from the top three models (see Table 1)

Model parameter	Model effect estimate (95% CI)	Relative variable importance	Percent of candidate models containing variable
Intercept	1.53 (0.61, 2.45)	—	—
Color (beige → blue‐green)	**−3.80 (−5.62, −1.98)**	1.00	75
Maculation (immaculate → spotted)	**3.40 (1.66, 5.15)**	1.00	75
Size (large robin → small cowbird)	**2.23 (0.70, 3.77)**	1.00	75
Color × size	**−3.33 (−5.89, −0.77)**	0.88	25
Maculation × size	−0.45 (−3.60, 0.91)	0.41	25
Color × maculation	0.12 (−1.70, 2.95)	0.27	25

Confidence intervals that do not overlap zero are noted in bold. For all models in Table 1, Nest ID and Study ID (i.e., this study or Rothstein) were included as random effects.

## 4 DISCUSSION

### 4.1 American robins’ responses to egg background color, maculation, and size

**Figure 3:**
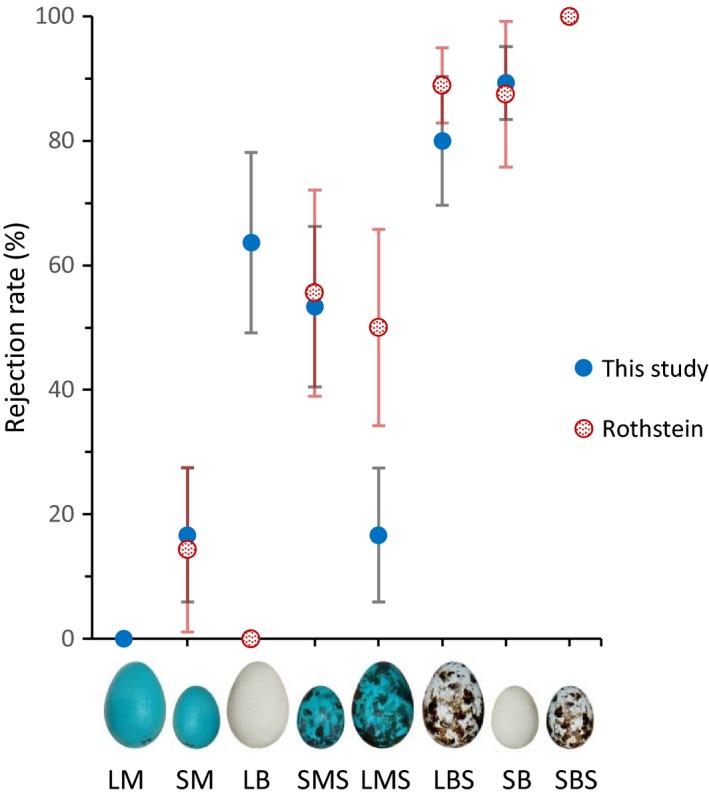
Rejection rates of experimental eggs placed in American robin nests separated by study (this study vs. Rothstein, 1982). Error bars represent approximate binomial standard error of rejection rates $\left(\surd \frac{\#\text{rejections}*(1-\#\text{rejections})}{\#\text{trials}}\right)$. Experimental egg treatment codes are in order of size (*L* = large robin‐sized, *S* = small cowbird‐sized), color (*M* = mimetic robin, *B* = beige cowbird), and maculation (*S* = spotted). Points overlap for *LM* (0%) and *SBS* (100%) where rejection rates were the same between studies

Similar to Rothstein's (1982) results, our study found that American robins responded to model egg features of background color, size, and maculation and were more likely to reject a model egg when it deviated from natural robin egg appearance toward natural cowbird egg appearance by at least two features (Table 2 and Figure 2). Results from the combined dataset of the two studies also confirmed that robin rejection decisions are best predicted by model egg background color and maculation, relative to model egg size. Additionally, model egg size and background color together influenced robins’ egg rejection responses (Tables 1 and 2); consistent with Rothstein's (1982) conclusion that robins may have a “tolerance” for eggs which vary in color or size alone, but will predictably reject model eggs which differ from natural robin egg appearance in both size and color (Figures 2 and 3). Because natural robin eggs can be quite variable in both size and color (Croston & Hauber, 2015b), tolerance of eggs varying in one of these two features alone may reduce possibility of mistakenly rejecting some of their own eggs (Rothstein, 1982).

### 4.2 Differences from Rothstein's (1982) findings

However, in contrast to Rothstein's (1982) conclusion that differences in at least two egg features are required to evoke any egg rejection responses in robins, the combined data revealed that robins do indeed recognize and reject foreign eggs that differ from their own eggs by a single feature toward the appearance of a cowbird egg. Specifically, model eggs differing from natural robin egg appearance in background color, maculation, or size alone were rejected at considerable rates compared to the complete absence of rejection responses toward completely robin‐mimetic control model eggs used for our own experiments (Figures 2 and 3, *LM*). Maculated model eggs increased rejection to 68% (Figure 2, *LMS*), model eggs with background color resembling cowbird egg color increased rejection to 37% (Figure 2, *LB*), and small cowbird‐sized model eggs increased rejection to 16% (Figure 2, *SM*). The greatest difference between robins’ responses to model eggs within a replicated treatment used by this study and that of Rothstein (1982) was for large cowbird‐colored model eggs: 37% rejection in this study and 0% rejection in Rothstein's (1982) (combined data Figure 2, *LB*; see Figure 3 *LB* for rejection response difference between separated study treatments). This disparity is likely due to the different paint colors used by this study and Rothstein (1982) to simulate natural cowbird egg background color, and possibly the smaller sample size (*n *=* *8) for this model egg treatment in Rothstein (1982) (Figure 3, egg treatment *LB*). Rothstein (1982) used white paint, chosen using Munsell color chips (Munsell, 1965), to simulate natural cowbird egg background color, whereas we used beige paint that generally matches natural cowbird egg background avian‐visible reflectance spectra (see Croston & Hauber, 2014 “BHCO ground” for details).

Rothstein (1982) originally concluded that robin egg rejection is guided by comparing an internal representation of natural robin eggs (an own egg “template”) with foreign eggs and is not specifically tuned toward rejection of cowbird‐like eggs. However, recent work suggests that robins do have substantial specificity in their egg recognition thresholds toward an intolerance of cowbird eggs. Hanley et al. (2017) demonstrated robins’ rejection decisions are fine‐tuned to the gradient of natural egg colors, but robins ignore perceivable differences along artificial color gradients, a finding inconsistent with the internal “own egg versus foreign egg” template (or multiple threshold, sensu Hanley et al., 2017) hypothesis. Similarly, Dainson, Hauber, López, Grim, and Hanley (2017) also found that robin egg rejection responses to egg spot coloration are likely tuned to a gradient of natural egg color patterns, where robins are more inclined to reject model eggs that have highly contrasting brown spots against a mimetic blue‐green robin egg background color.

In summary, Rothstein's (1982) benchmark study set the standard for research on host recognition of brood parasite eggs. Since its publication, a vast amount of methodologically similar work has investigated which egg features evoke foreign egg rejection behavior in many different avian brood parasite–host species (de la Colina, et al., 2012; Croston & Hauber, 2014; López‐de‐Hierro & Moreno‐Rueda, 2010; Moskát, et al., 2008; Segura, Di Sallo, Mahler, & Reboreda, 2016; Underwood & Sealy, 2006b). Generally, the relative difference between own and foreign egg background coloration seems to be the most important determinant of whether hosts accept or reject foreign eggs (Cassey, et al., 2008; Hauber et al., 2015; Moskát et al., 2008; Spottiswoode & Stevens, 2010).

### 4.3 Future directions for egg recognition research

Here, we confirmed that discrete, categorical differences in egg background color, maculation, and size are all important cues for foreign egg recognition in robins. However, recent experimental approaches have set a new standard, using model eggs, which vary continuously rather than discretely, along natural gradients of different background colors (Hanley et al., 2017), as well as maculation patterns and contrasts (Dainson et al., 2017), and sizes and shapes (Igic et al., 2015). Egg rejection studies performed with continuously varying model eggs, in combination with avian visual modeling (Avilés, 2008; Cassey et al., 2008; Spottiswoode & Stevens, 2010), allow for estimation of perceivable differences to the host species of interest for each model egg feature. Thus, they may provide more robust, meaningful comparisons of the relative influence of each separate egg feature for a given host species’ egg recognition threshold than studies using model eggs which vary only discretely. Given the recent and rapid development of 3D‐printing to construct model eggs of differing shapes and sizes for use in artificial brood parasitism experiments (Igic et al., 2015), and our knowledge of the avian‐visible egg color, maculation, and pigment diversity (Hanley, Grim, Cassey, & Hauber, 2015), it is now certainly possible to design such a suite of experiments for other brood parasite–host species, like those already performed with robins (Dainson et al., 2017; Hanley et al., 2017).

## CONFLICT OF INTEREST

The authors declare they have no conflict of interests.

## AUTHOR CONTRIBUTIONS

Alec Luro prepared the manuscript, and performed experiments and data analysis. data analyses. Branislav Igic, Rebecca (Beki) Croston, and Analia V. Lopez contributed to manuscript drafts, performed the experiments, and collected the data. Matthew D. Shawkey and Mark E. Hauber conceived and designed the work. All authors contributed to edited drafts of the manuscript.

## ETHICAL APPROVAL

All experiments and procedures of this study were IUACUC‐approved (MH 2/16‐T3) and complied with U.S. laws.

## DATA AVAILABILITY

All data generated or analyzed during this study are included in this published article [and its Table S1].
